# Supplementary material for: The effects of group adaptation on task performance: An agent-based approach
Source: PLoS One. 2023 Aug 28;18(8):e0290578. doi: 10.1371/journal.pone.0290578 (PMC10461837; doi:10.1371/journal.pone.0290578)
Supplement: S1 Appendix — (PDF) [file pone.0290578.s001.pdf]

A. Notation

Table 1. Notation

Notation	Description
N	Number of decisions
K	Number of interdependencies between decisions
M	Number of subtasks / agents in the group
P	Total number of agents
I	Number of solutions known by an agent
$C(\cdot)$	Performance function
$U(\cdot)$	Utility function
$\frown$	Concatenation operator
τ	Periods between two rounds of group adaptation
$\mathbb{P}$	Learning probability
t	Time steps
$\mathbf{d}$	Vector of binary decisions
d_n	Decision n
c_n	Performance contribution of decision n
$\overline{C}_t$	Normalized performance in t
C^*	Maximum achievable performance
$\mathbf{d}_t$	Solution to the entire task implemented in t
$\mathbf{S}_{mt}$	All solutions known by agent m to solve the assigned subtask at time t
$\hat{\mathbf{d}}_{mi}$	i^{th} solution known by agent m to solve the assigned subtask
$\hat{\mathbf{d}}^*_{mt}$	Utility-maximizing solution known by agent m to solve the assigned subtasks in t
$\mathbf{d}_{mt}$	Solution implemented by agent m to solve their subtask in t
$\mathbf{D}_{mt}$	Solutions implemented by agents other than m to solve their subtask in t
$\mathbf{X}$	Set of independent variables
$\mathbf{X}^s$	Set of independent variables in scope of the analysis
$\mathbf{X}^c$	Complementary set of independent variables

B. Data analysis

We use simulated data to train regression neural networks and analyze the data following the procedure introduced in Section 4. Table 3 gives an overview of the used datasets and the corresponding parameter settings, the trained models (type neural network used in the regression and number of nodes), and RMSE and R^2. Since we are interested in the average effects, all models include data for periods $t = \{1, \ldots, 100\}$. For details about the parameters included in the trained regression model, see Table 1.

We trained the regression neural networks in the Matlab Regression Learner App, which returns a number of models for every dataset. Out of the models, we selected those with the lowest RMSE (root-mean-square error) and highest R^2 for our analysis.

Table 2. Regression analyses

Parameters				Neural Network		Validation	
K	*Matrix*	τ	$\mathbb{P}$	Type	Nodes	RMSE	R^2
**Section 5.1:**							
{3,5}	all	∞	$\{0:0.1:1\}$	Wide	100	0.0050	0.99
**Section 5.2:**							
3	Block	∞	$\{0:0.1:1\}$	Wide	100	0.0017	1
5	Block	∞	$\{0:0.1:1\}$	Wide	100	0.0018	1
3	Block	10	$\{0:0.1:1\}$	Wide	100	0.0047	1
5	Block	10	$\{0:0.1:1\}$	Wide	100	0.0030	1
3	Block	1	$\{0:0.1:1\}$	Wide	100	0.0024	1
5	Block	1	$\{0:0.1:1\}$	Wide	100	0.0020	1
**Section 5.3:**							
3	all	∞	0	Wide	100	0.0136	0.87
5	all	∞	0	Tri-layered	10-10-10	0.0073	0.79
3	all	10	0	Wide	100	0.0139	0.78
5	all	10	0	Tri-layered	10-10-10	0.0094	0.86
3	all	1	0	Wide	100	0.0129	0.78
5	all	1	0	Tri-layered	10-10-10	0.0062	0.91
**Section 5.4:**							
3	all	∞	$\{0:0.1:1\}$	Wide	100	0.0025	1
5	all	∞	$\{0:0.1:1\}$	Wide	100	0.0028	1
3	all	10	$\{0:0.1:1\}$	Wide	100	0.0052	0.99
5	all	10	$\{0:0.1:1\}$	Wide	100	0.0039	1
3	all	1	$\{0:0.1:1\}$	Wide	100	0.0033	1
5	all	1	$\{0:0.1:1\}$	Wide	100	0.0031	1

C. Validation and robustness analysis

In this paper, we employ agent-based modelling and simulation not to empirically validate a theory but rather to gain better theoretical insights into the topic of group adaptation [1]. Following [2], we provide two ways to check the validity of the results: *(i)* we discuss the main assumptions of the model in Section 3 and *(ii)* we check the robustness of the results against variations in the population size P and the number of decisions N in the following subsection.

C..1 Effects in smaller populations

In this subsection, we consider a small population (i.e., $P = 6$) and compare the effects of group adaptation, individual learning, and task complexity on performance with those outlined in Section 5.1. In a scenario with a small population, each subtask has only two assigned agents. This implies that the group can only turn to one agent (outside the current group)per subtask when looking for new members.

In Table 4, we provide an overview of the regression analyses performed for a small population size. We present the overall effects between population size and performance

for small but also large (i.e., $P = 30$) population sizes in Fig 7A-D. Figs 8 and 9 show the partial dependencies between task performance and the individual learning probability and the interdependence structure, respectively, for a small population size. Finally, we present the results for a simultaneous variation in the individual learning probability and interdependence structure for a small population size in Fig 10.

Table 3. Regression analyses: $P = 6$

Parameters				Neural Network		Validation	
K	*Matrix*	τ	$\mathbb{P}$	Type	Nodes	RMSE	R^2
**Overall effects:**							
{3,5}	all	∞	$\{0:0.1:1\}$	Wide	100	0.0044	0.99
**Learning:**							
3	Block	∞	$\{0:0.1:1\}$	Wide	100	0.0021	1
5	Block	∞	$\{0:0.1:1\}$	Wide	100	0.0018	1
3	Block	10	$\{0:0.1:1\}$	Wide	100	0.0010	1
5	Block	10	$\{0:0.1:1\}$	Wide	100	0.0013	1
3	Block	1	$\{0:0.1:1\}$	Wide	100	0.0018	1
5	Block	1	$\{0:0.1:1\}$	Wide	100	0.0015	1
**Interdependence structure:**							
3	all	∞	0	Wide	100	0.0010	1
5	all	∞	0	Wide	100	0.0010	1
3	all	10	0	Wide	100	0.0010	1
5	all	10	0	Wide	100	0.0010	1
3	all	1	0	Wide	100	0.0010	1
5	all	1	0	Wide	100	0.0010	1
**Simultaneous effects:**							
3	all	∞	$\{0:0.1:1\}$	Wide	100	0.0024	1
5	all	∞	$\{0:0.1:1\}$	Wide	100	0.0028	1
3	all	10	$\{0:0.1:1\}$	Wide	100	0.0028	1
5	all	10	$\{0:0.1:1\}$	Wide	100	0.0033	1
3	all	1	$\{0:0.1:1\}$	Wide	100	0.0027	1
5	all	1	$\{0:0.1:1\}$	Wide	100	0.0028	1

Fig 7. Comparison of overall effects between population sizes.

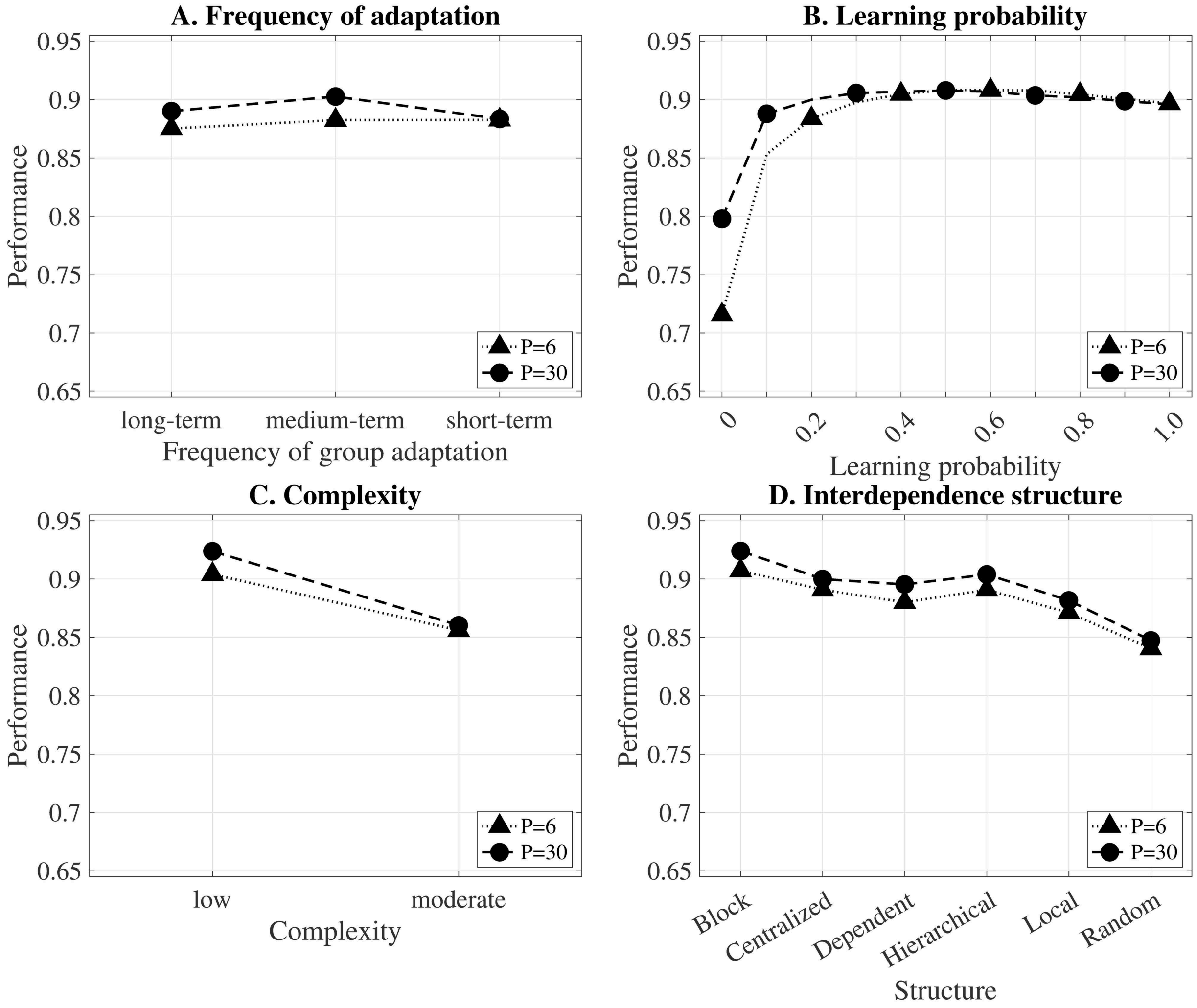

Fig 8. Partial dependencies between task performance and the learning probability for a small population size (P=6).

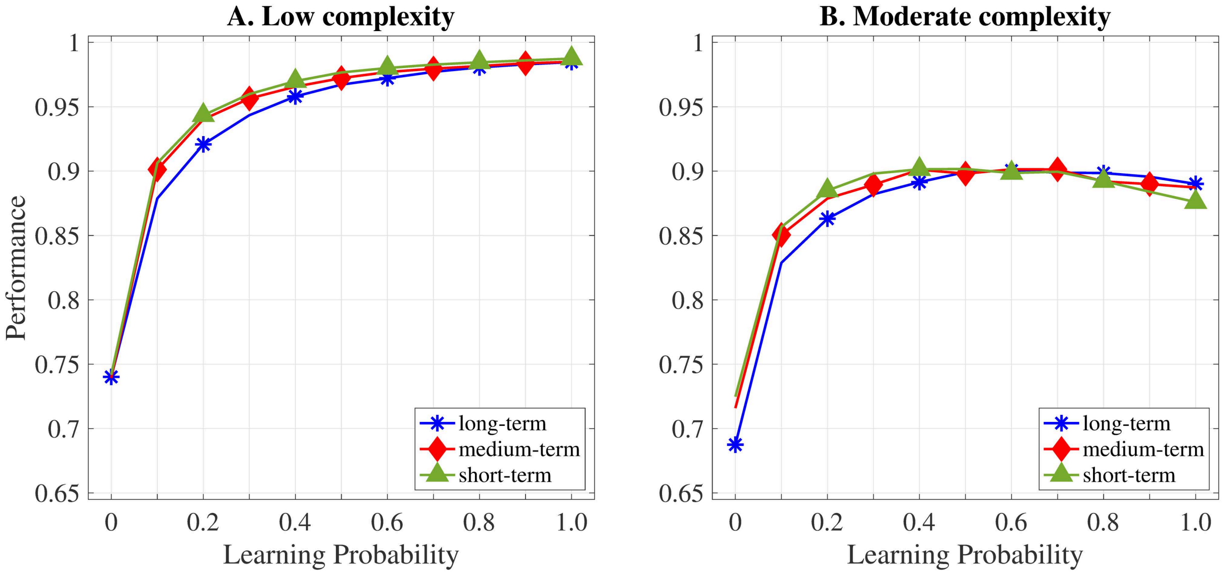

Fig 9. Partial dependencies between task performance and interdependence structures for a small population size (P=6).

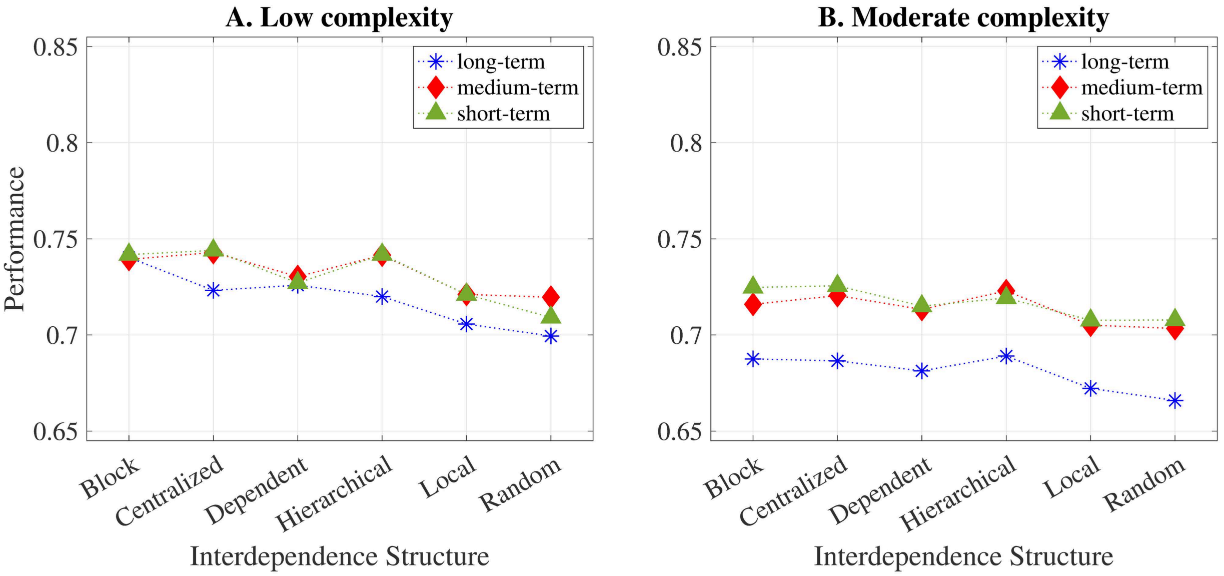

Fig 10. Partial dependencies for a simultaneous variation of moderating factors for a small population size (P=6).

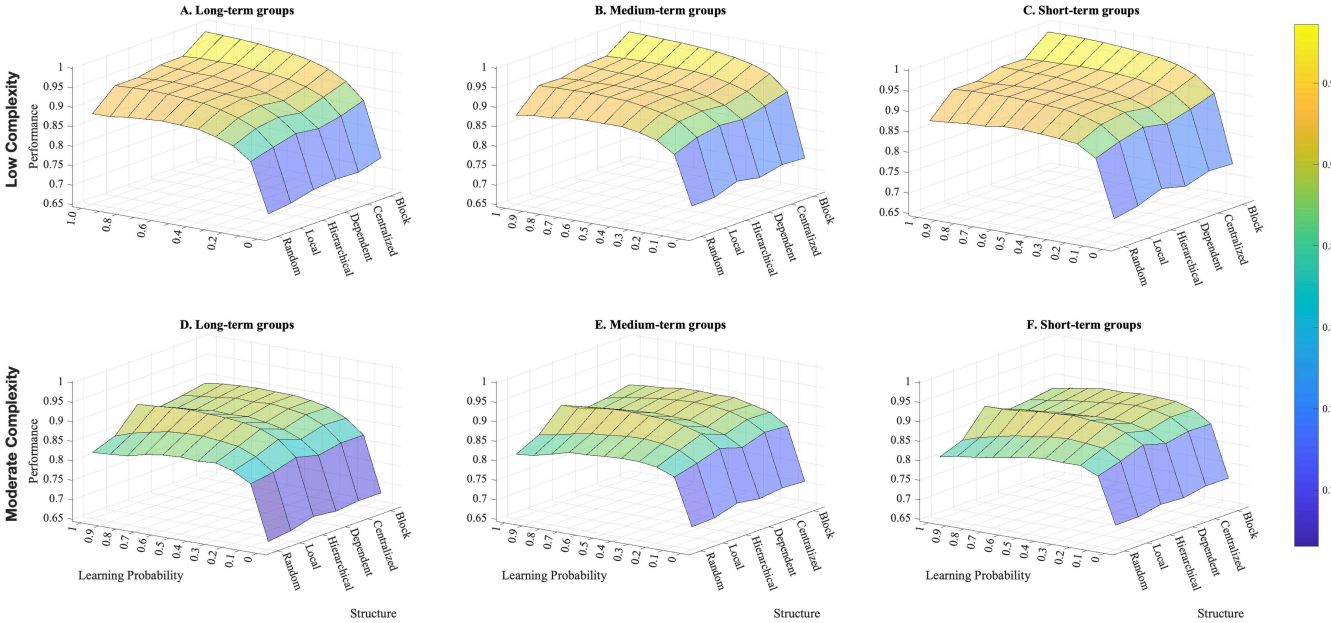

Our robustness analysis shows that, while the patterns are similar, some differences depending on population size are worth a discussion. First, we show that a large

population achieves higher average performances than a small population at lower levels of individual learning. These differences decrease as $\mathbb{P}$ grows. Second, there are differences in the patterns of individual learning and the interdependence structure. Results for individual learning show that, in tasks of moderate complexity (see Fig 8B), the negative effects of individual learning on the performance of short-term groups are much more relevant for larger than smaller populations (see Fig 4B for the results for larger population sizes). If the population is smaller, the group has fewer alternatives to choose from to replace group members, which reduces the risk for overexploration [3]. Regarding the interdependence structure, results for low (see Fig 9A) and moderate complexity (see Fig 9B) show that, while the patterns remain similar, the overall effects of the interdependence structure are less relevant for smaller than for larger population sizes (see also Fig 5).

C..2 Effects in tasks with a lower number of decisions

In this subsection, we consider a task with a lower number of decisions (i.e., $N = 6$) and compare the general effects of group adaptation, individual learning, and task complexity on performance with those outlined in Section 5.1 (i.e., for $N = 12$). In this scenario we reduce K proportionally. Consequently, if the task complexity is low (i.e., $K = 1$), one decision affects its own contribution and the contribution of one other decision. If the task is moderately complex (i.e., $K = 2$), one decision affects its own contribution and the contribution of two other decisions. We hold the remaining variables constant, so each of the $M = 3$ subtasks consists of two decisions $S = 2$. Fig 11 shows the interdependence structures considered for this new configuration.

[Fig 11 near here]: Interdependence structures for a task with a lower number of decisions ($N = 6$). Interdependencies between the performance contributions (represented on the y-axes) and the decisions (represented on the x-axes) are indicated with an x. Each contribution depends on its own decision (see Equation 1), so there is an x in each element along the main diagonal. Solid lines indicate subtasks assigned to agents.

Block diagonal

(A) Low Complexity (K=1)

Contributions \ Decisions	1	2	3	4	5	6
1	x	x	-	-	-	-
2	x	x	-	-	-	-
3	-	-	x	x	-	-
4	-	-	x	x	-	-
5	-	-	-	-	x	x
6	-	-	-	-	x	x

(B) Moderate complexity (K=2)

Contributions \ Decisions	1	2	3	4	5	6
1	x	x	x	-	-	-
2	x	x	x	-	-	-
3	x	x	x	-	-	-
4	-	-	-	x	x	x
5	-	-	-	x	x	x
6	-	-	-	x	x	x

Local

(A) Low Complexity (K=1)

Contributions \ Decisions	1	2	3	4	5	6
1	x		-	-	-	x
2	x	x	-	-	-	-
3	-	x	x		-	-
4	-	-	x	x	-	-
5	-	-	-	x	x	-
6	-	-	-	-	x	x

(B) Moderate complexity (K=2)

Contributions \ Decisions	1	2	3	4	5	6
1	x	-	-	-	x	x
2	x	x	-	-	-	x
3	x	x	x	-	-	-
4	-	x	x	x	-	-
5	-	-	x	x	x	-
6	-	-	-	x	x	x

Random

(A) Low Complexity (K=1)

Contributions \ Decisions	1	2	3	4	5	6
1	x	-	-	x	-	-
2	x	x	-	-	-	-
3	-	-	x	-	-	x
4	-	-	x	x	-	-
5	-	x	-	-	x	-
6	-	-	-	-	x	x

(B) Moderate complexity (K=2)

Contributions \ Decisions	1	2	3	4	5	6
1	x	x	-	-	-	x
2	-	x	x	-	x	-
3	x	-	x	x	-	-
4	-	x	-	x	-	x
5	x	-	x	-	x	-
6	-	-	-	x	x	x

Centralised

(A) Low Complexity (K=1)

Contributions \ Decisions	1	2	3	4	5	6
1	x	x	-	-	-	-
2	x	x	-	-	-	-
3	x	-	x	-	-	-
4	x	-	-	x	-	-
5	x	-	-	-	x	-
6	x	-	-	-	-	x

(B) Moderate complexity (K=2)

Contributions \ Decisions	1	2	3	4	5	6
1	x	x	x	-	-	-
2	x	x	x	-	-	-
3	x	x	x	-	-	-
4	x	x	-	x	-	-
5	x	x	-	-	x	-
6	x	x	-	-	-	x

Dependent

(A) Low Complexity (K=1)

Contributions \ Decisions	1	2	3	4	5	6
1	x	-	-	-	-	-
2	-	x	-	-	-	-
3	-	-	x	-	-	-
4	-	-	-	x	-	-
5	-	-	-	-	x	x
6	x	x	x	x	x	x

(B) Moderate complexity (K=2)

Contributions \ Decisions	1	2	3	4	5	6
1	x	-	-	-	-	-
2	-	x	-	-	-	-
3	-	-	x	-	-	-
4	-	-	-	x	x	x
5	x	x	x	x	x	x
6	x	x	x	x	x	x

Hierarchical

(A) Low Complexity (K=1)

Contributions \ Decisions	1	2	3	4	5	6
1	x	-	-	-	-	-
2	x	x	-	-	-	-
3	x	x	x	-	-	-
4	x	-	-	x	-	-
5	x	-	-	-	x	-
6	x	-	-	-	-	x

(B) Moderate complexity (K=2)

Contributions \ Decisions	1	2	3	4	5	6
1	x	-	-	-	-	-
2	x	x	-	-	-	-
3	x	x	x	-	-	-
4	x	x	x	x	-	-
5	x	x	x	-	x	-
6	x	x	x	-	-	x

In Table 5, we provide an overview of the regression analyses performed for a task with a lower number of decisions. Fig 12A-D shows the general effects for the two task sizes considered. In Fig 13A-B, we present the partial dependencies between task performance and individual learning for tasks with a lower number of decisions. Fig 14A-B includes the partial dependencies between task performance and the task interdependence structure. Finally, Fig 15 presents the partial dependencies for a simultaneous variation in the moderating factors.

Table 4. Regression analyses: $N = 6$

Parameters				Neural Network		Validation	
K	*Matrix*	τ	$\mathbb{P}$	Type	Nodes	RMSE	R^2
**Overall effects:**							
{1,2}	all	∞	$\{0 : 0.1 : 1\}$	Wide	100	0.0041	0.99
**Learning:**							
1	Block	∞	$\{0 : 0.1 : 1\}$	Wide	100	0.0016	1
2	Block	∞	$\{0 : 0.1 : 1\}$	Wide	100	0.0012	1
1	Block	10	$\{0 : 0.1 : 1\}$	Wide	100	0.0013	1
2	Block	10	$\{0 : 0.1 : 1\}$	Wide	100	0.0014	1
1	Block	1	$\{0 : 0.1 : 1\}$	Wide	100	0.0010	1
2	Block	1	$\{0 : 0.1 : 1\}$	Wide	100	0.0016	1
**Interdependence structure:**							
1	all	∞	0	Wide	100	0.0010	1
2	all	∞	0	Wide	100	0.0010	1
1	all	10	0	Wide	100	0.0010	1
2	all	10	0	Wide	100	0.0010	1
1	all	1	0	Wide	100	0.0010	1
2	all	1	0	Wide	100	0.0010	1
**Simultaneous effects:**							
1	all	∞	$\{0 : 0.1 : 1\}$	Wide	100	0.0020	1
2	all	∞	$\{0 : 0.1 : 1\}$	Wide	100	0.0028	1
1	all	10	$\{0 : 0.1 : 1\}$	Wide	100	0.0019	1
2	all	10	$\{0 : 0.1 : 1\}$	Wide	100	0.0033	1
1	all	1	$\{0 : 0.1 : 1\}$	Wide	100	0.0029	1
2	all	1	$\{0 : 0.1 : 1\}$	Wide	100	0.0028	1

Fig 12. Comparison of overall effects between task sizes.

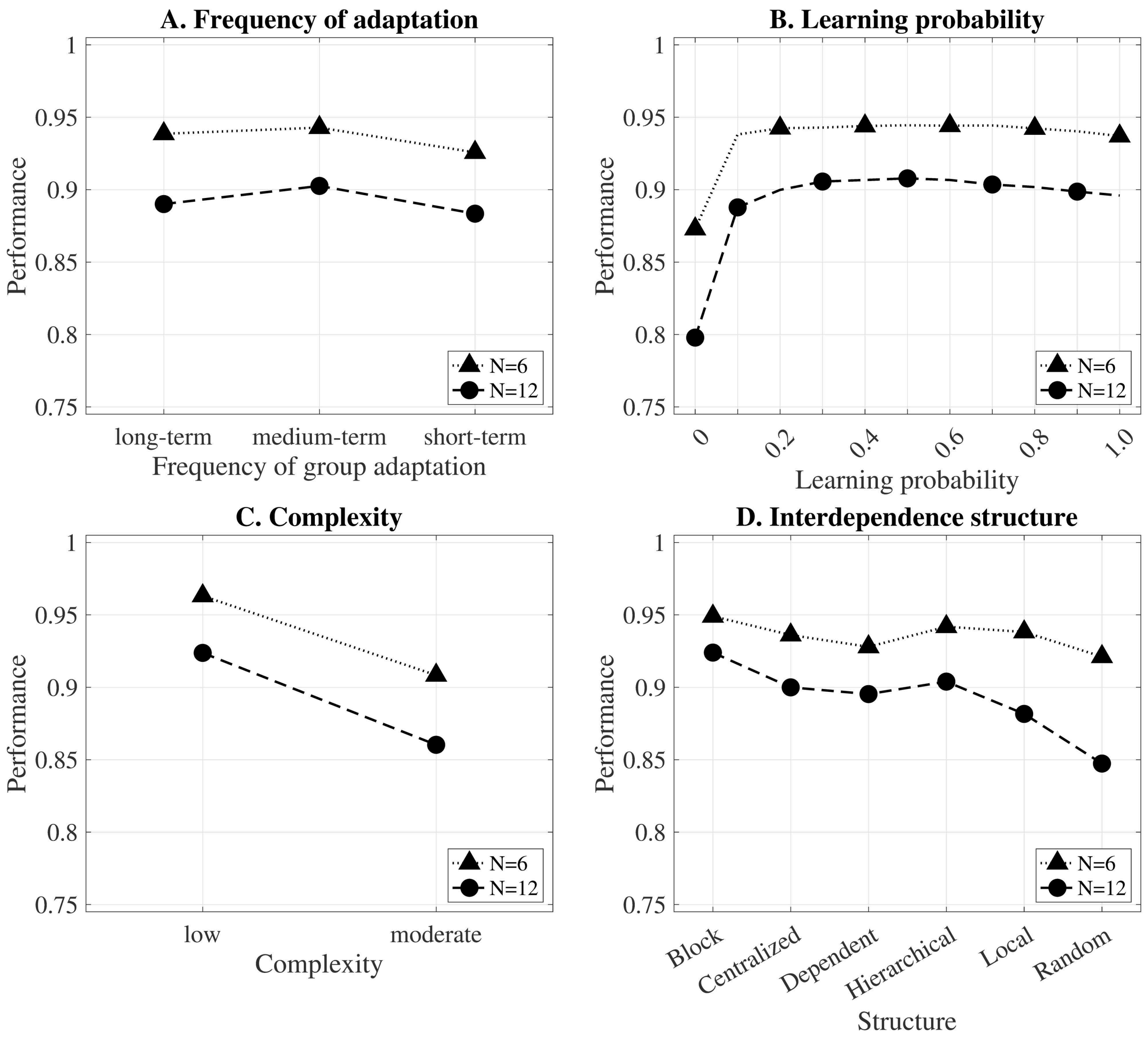

Fig 13. Partial dependencies between task performance and the learning probability in a task with a lower number of decisions (N=6).

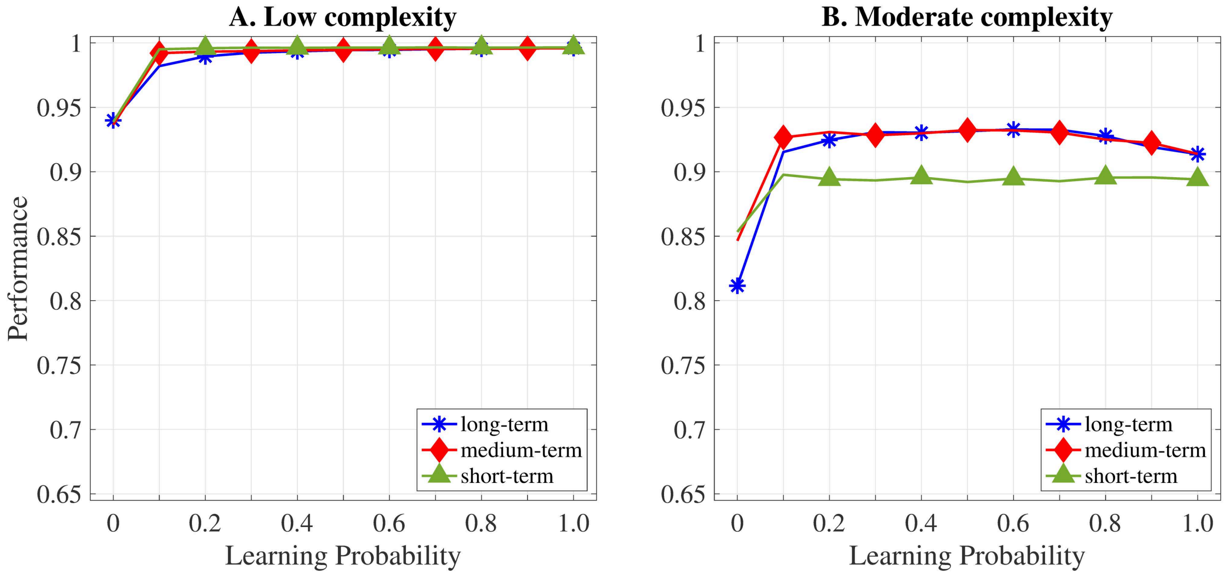

Fig 14. Partial dependencies between task performance and the interdependence structures in a task with a lower number of decisions (N=6).

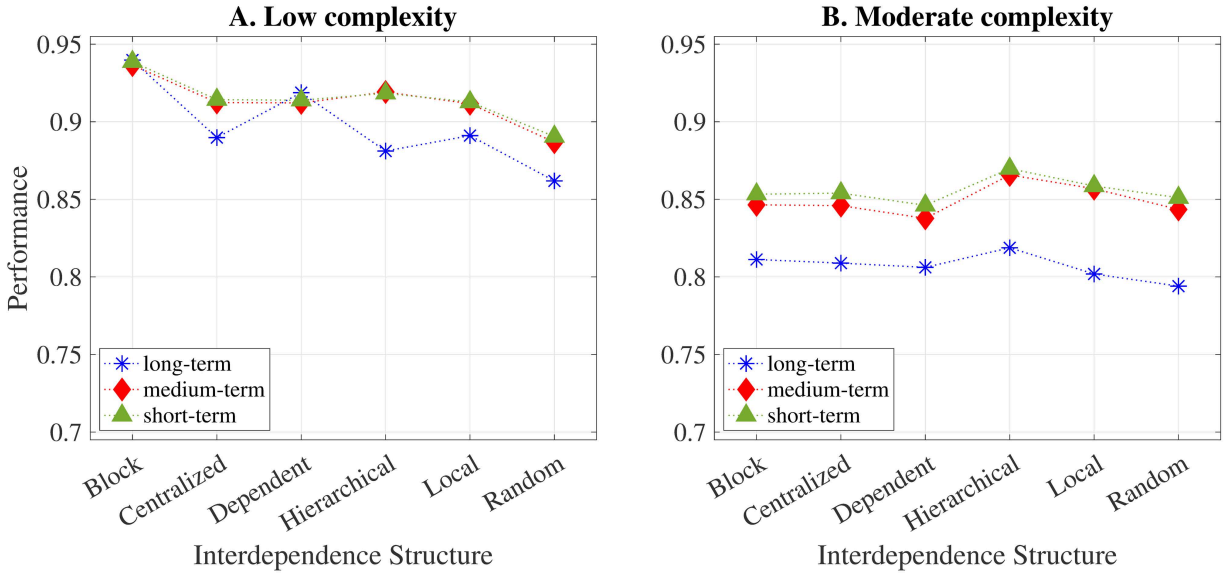

Fig 15. Partial dependencies for a simultaneous variation of moderating factors in a task with a lower number of decisions (N=6).

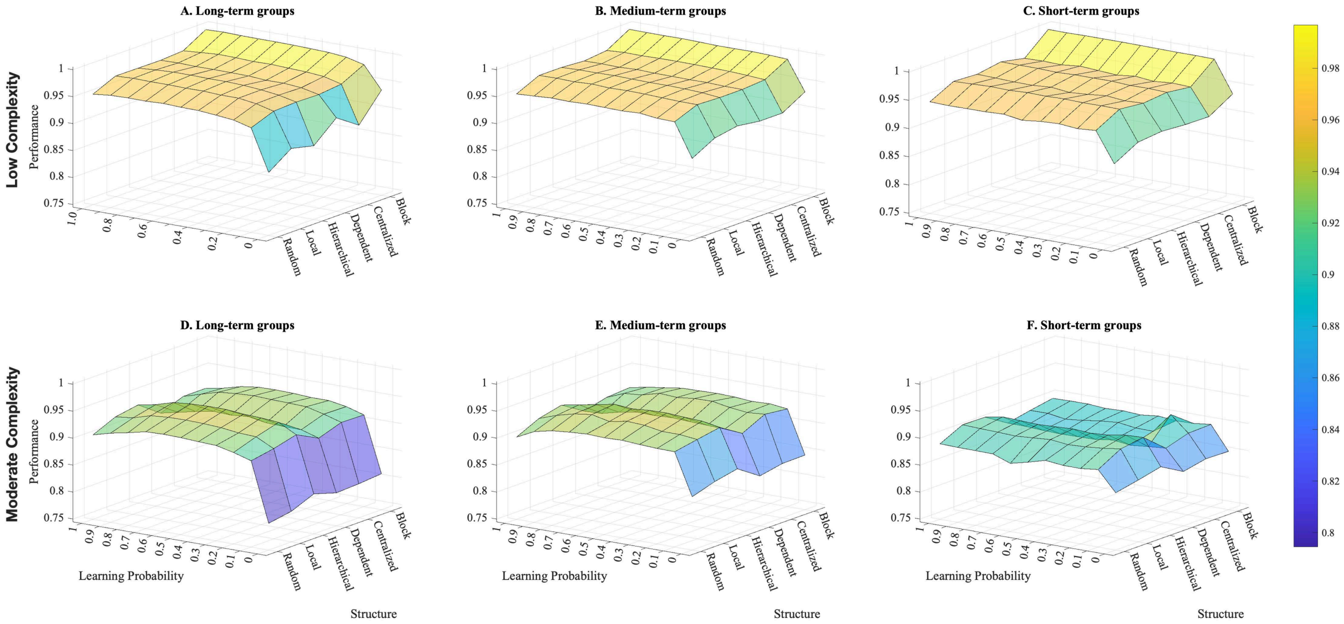

Regarding the general effects of group adaptation, individual learning, and task complexity, Fig 12A-C shows that the patterns are very similar for both task sizes. Regarding individual learning, for low complexity (see Fig 13A), we observe a very similar pattern for short- and medium-term groups. Performance increases significantly when agents start to learn, but further increases in learning barely affect task performance. In tasks of moderate complexity (see Fig 13B) the patterns are similar irrespective of the number of decisions for long- and medium-term groups. Increasing $\mathbb{P}$

improves task performance with decreasing marginal effects, until the effect turns slightly negative.

We should note, however, that there are some differences in the results depending on the number of decisions of the task. First, groups perform significantly better if there are less decisions in the task. This result is intuitive: If there are less decisions, there are less possible solutions to the task. Consequently, finding the optimal solution to the task becomes easier [4]. Second, for short-term groups, we do not see the negative effect of individual learning that exists for $N = 12$ in tasks of moderate complexity (see Fig 4). Instead, performance stabilizes for $\mathbb{P} > 0.1$ (see Fig 13B). Third, in a task with a lower number of decisions, a long-term group's performance is similar to that of short-and medium-term groups if complexity is low (see Fig 13A) and to that of medium-term groups if task complexity is moderate (see Fig 13B). By contrast, in a task with a higher number of decisions, long-term groups perform worse at lower levels of $\mathbb{P}$ (see Fig 4). Finally, in Fig 12D we see that the performance is more sensitive to the interdependence structure, when the number of decisions is high. This is supported by the differentiated results shown in Fig 14A-B. This result is intuitive, too: As the number of decisions increases, tasks become more interdependent and the interdependence structure becomes more relevant [5].

References

1. Wall F, Leitner S. Agent-based computational economics in management accounting research: Opportunities and difficulties. Journal of Management Accounting Research. 2021;33(3):189–212.

2. Law AM. Simulation Modeling & Analysis. 5th ed. New York, NY, USA: McGraw-Hill; 2015.

3. Bunderson JS, Sutcliffe KM. Management team learning orientation and business unit performance. Journal of Applied Psychology. 2003;88(3):552–560.

4. Levinthal DA. Adaptation on rugged landscapes. Management Science. 1997;43(7):934–950.

5. Rivkin JW, Siggelkow N. Patterned interactions in complex systems: Implications for exploration. Management Science. 2007;53(7):1068–1085.
