# Supplementary material for: The effects of group adaptation on task performance: An agent-based approach
Source: PLoS One. 2023 Aug 28;18(8):e0290578. doi: 10.1371/journal.pone.0290578 (PMC10461837; doi:10.1371/journal.pone.0290578)
Supplement: Attachment — Submitted filename: Comments.docx [file pone.0290578.s005.docx]

Date :2023-06-30

**Reviewer Comments**

The selection of research topic **PONE-D-23-09718, "The effects of group adaptation on task performance: An agent-based approach**” is very advanced and more applicable to existing scenarios. The author has attempted to describe the entire research intervention of a data-driven support model for group adoption and learning. During the process of reviewing, I found that there is merit in the research paper, and I have been accepted for publication by cross-checking and adding the following:

1. Describe your model structure in a sequential, usual manner. State the random variable.

2. Group adoption of the task: This will depend on many attributes, such as whether the intelligent individual has adopted the task at an early stage, what measurement/ parameters you will consider for late adopters, and how to estimate its likelihood.

3.How does individual learning moderate the relationship between group adaptation and task performance? In this criteria,  group adoption will depend on the interdependence among the many work policies. Please describe what measures you have taken to correlate the above stature.

4.How does complexity moderate the relationship between group adaptation and Task performance? The above criteria clearly depend on the above two indicators, as mentioned.

**Dr Basavarajaiah DM**

**Editor –Academic**
